# Systemic Treatment of Gastroenteropancreatic Neuroendocrine Carcinoma

**DOI:** 10.1007/s11864-021-00866-9

**Published:** 2021-06-10

**Authors:** Kazhan Mollazadegan, Staffan Welin, Joakim Crona

**Affiliations:** Department of Medical Sciences, Uppsala University, Akademiska Sjukhuset ing 100, 75185 Uppsala, Sweden

**Keywords:** Systemic treatment, Extra pulmonary, Poorly differentiated, High-grade, Gastroenteropancreatic carcinoma, Neuroendocrine carcinoma

## Abstract

Treatment recommendations for advanced gastroenteropancreatic neuroendocrine carcinomas (GEP-NEC) are based on uncontrolled, mainly retrospective data. Chemotherapy can offer palliative relief, but long-lasting complete responses or cures are rare. The European Neuroendocrine Tumour Society (ENETS) and European Society for Medical Oncology (ESMO) recommend platinum-based chemotherapy as first-line treatment. This has been the golden standard since the late 1980s and has been evaluated in mostly retrospective clinical studies. However, progression is inevitable for most patients. Unfortunately, data on effective second-line treatment options are scant, and ENETS and ESMO recommendations propose fluorouracil- or temozolomide-based chemotherapy schedules. As such, there is a huge unmet need for improved care. Improved knowledge on GEP-NEC biology may provide a pathway towards more effective interventions including chemotherapy, targeted gene therapy, peptide receptor radionuclide therapy, as well as immune checkpoint inhibitors. The review summarises this current state of the art as well as the most promising developments for systemic therapy in GEP-NEC patients.

## Introduction

Gastroenteropancreatic neuroendocrine carcinomas (GEP-NECs) are highly aggressive cancers associated with poor prognosis and fast disease progression. The incidence of this group of diseases is thought to have increased over the last 3–4 decades and is now reported to be about 7 per 100,000 persons/year [1–6]. The classification has evolved throughout the years based on better understanding of disease biology as well as improved diagnostic tools. Since 2017, the World Health Organization (WHO) classifications [7, 8] recognise poorly differentiated high-grade NEC as a distinct entity from high-grade neuroendocrine tumours (NET) [9]. This is now reflected in treatment recommendations where first-line platinum-based chemotherapy is reserved for NECs and not for high-grade NETs [10, 11]. Despite this improved understanding of NECs, available systemic therapies are considered to provide only palliative relief, and recommendations are mostly supported by uncontrolled, retrospective studies [12••, 13]. New therapies are emerging, with both immune checkpoint inhibitors and genetic-guided therapy having shown some interesting results. One promising example is treatment with anti-*BRAF* therapy in colorectal NEC displaying oncogenic *BRAF* mutations [14, 15].

In this review article, we aim to provide an overview of the current recommendations for systemic therapy in GEP-NEC. Based on a comprehensive review of biomedical and clinical trials databases, we present the evidence underlying available recommendations, with focus on first-line and second-line scenarios. Finally, we aim to review the current state of the art and research directions that can improve treatment options for this high-risk patient group.

## Classification of neuroendocrine neoplasms

The classification and terminology of gastroenteropancreatic neuroendocrine neoplasms (GEP-NENs) have been revised and changed throughout history. The original classification for these diseases was based on embryological origin and recognised foregut, midgut, and hindgut variants [16, 17]. In 2000, a new classification was presented by the WHO that recognised well-differentiated and poorly differentiated endocrine carcinoma based on their distinct biological characteristics and clinical outcomes [18]. A decade later, demand for improved prognostic and diagnostic criteria led to the WHO 2010 classification [8] based on the Ki-67 proliferation index, mitotic index, and tumour cell morphology. This also introduced the term ‘NEN’ as an umbrella term for well and poorly differentiated tumours of neuroendocrine cell origin [19].

According to the 2010 WHO grading and classification [20], GEP-NENs are separated into well-differentiated NET of low or intermediate grade, with a Ki-67 value of <3% and 3–20%, respectively, and high-grade poorly differentiated NEC grade (G) 3 with Ki-67 >20%. GEP-NEC G3 is further divided histopathologically into large-cell or small-cell NEC [20–22]. In 2017, the grading and classification system was updated for pancreatic NENs and introduced the category of well-differentiated high-grade NET G3 with Ki-67 ≥20%. Since 2019, this is accepted for all gastrointestinal NENs [8].

Further categorisation of NEC can be made according to disease site, and the most important distinction is pulmonary or extrapulmonary NEC. Extrapulmonary NECs occur most commonly in the gastrointestinal tract but can also be found in the urinary bladder, cervix, and prostate [2]. GEP-NECs display high proliferation rates and are associated with a poor prognosis, even when diagnosed without metastases [23]. Most GEP-NEC patients present with distant metastases, and this disease state is considered to be incurable. For GEP-NEC patients that did not receive any treatment, survival has been reported to be as short as 1 month, while those patients that had received chemotherapy had a median survival of 12–19 months in various studies [9, 24].

## Biology of neuroendocrine neoplasms

The genetic landscape of GEP-NEC is incompletely characterised, but it is clear that mutations in *TP53*, *KRAS*, *PIK3CA/PTEN*, or *BRAF* play an important role in malignant transformation and progression (reviewed in [25, 26]). Girardi et al. [27] performed a systematic review on this topic and identified 33 relevant studies. Microsatellite instability was found in about 10% of gastric and colorectal NECs. Additionally, an impaired mismatch repair pathway has been observed in 93% (30/32) of colorectal NECs [28]. Chalmers et al. [29] were able to describe tumour mutational burden using targeted comprehensive genetic profiling of 100,000 human cancer genomes across 100 tumour types. NECs were also analysed in this study, and in 140 gastrointestinal NECs, the median tumour mutational burden was 3.7, and 4.3% had at least 20 mutations per megabase. Among 233 pancreatic NECs, the median tumour mutational burden was 2.7, and 1.7% of patients had at least 20 mutations per megabase. The results from the study indicated that high tumour mutational burden may correlate with a better response to immunotherapy.

Girardi et al. also showed how alterations in *TP53* gene and p53 protein was found to occur in most, if not all, GEP-NECs. Other common alterations were observed to involve the MAPK/ERK, p16/Rb/cyclin D1, and Hedgehog and Notch signalling pathways, with somatic alterations commonly observed in *KRAS, BRAF*, *RB1*, or *BCL2.* Furthermore, small-cell and large-cell NECs have been associated with different molecular signatures. Small-cell NEC-like tumours were characterised by the bi-allelic inactivation of both *TP53* and *RB1* [30, 31], whereas the genomics of large-cell NEC were more complex and heterogeneous having a ‘carcinoma-like’ signature, with similar profiles to those observed in carcinomas without neuroendocrine differentiation occurring in the corresponding location [30, 31].

## Current treatment strategies

Current ENETS (2016) [13] and ESMO (2020) guidelines [12••] for management of GEP-NECs take note of the extent and resectability of the disease as well as its proliferative activity to guide treatment decisions. The staging system for adenocarcinomas [32] should be used, and patients with localised disease can be considered for surgical resection followed by adjuvant chemotherapy. High-risk features such as large tumour size and advanced disease constitute contraindications for surgery. Instead, those patients should be considered for systemic therapy to provide palliative relief. Patients with NEC are thought to benefit from having their cases assessed by a multidisciplinary conference with experience of the disease group. However, it should not delay the initial patient management as early treatment onset is thought to be crucial to avoid rapid patient deterioration.

### First-line setting

Platinum-based chemotherapy using cisplatin/etoposide or carboplatin/etoposide is recommended as first-line therapy for advanced NEC in both ENETS and ESMO guidelines [12••,^13^]. The standard administration route is by intravenous infusion, though oral etoposide was recently reported to have equivalent efficacy and also reduced hospital stay from 3 days to 1 [33].

The concept of platinum-based chemotherapy for treatment of NEC originated from the experience in small-cell lung cancer which shares many biological characteristics with NEC [34]. In 1991, Moertel et al. [10] reported on forty-five patients with metastatic NET that received cisplatin/etoposide. Among 18 patients with ‘anaplastic NEC’, nine had partial response (PR), three had complete response (CR), and the median duration of response was 8 months. However, in 27 patients with well-differentiated morphology, the response rate (RR) was only 7%. This was validated by Mitry et al. [11] who studied response to cisplatin/etoposide in 53 patients with ‘NETs’ (according to the 1980s Gould and Warren classifications [35,36]). Twelve patients had well-differentiated and 41 had poorly differentiated tumours. The RR among poorly differentiated cases was 41%, while only 8% of well-differentiated tumours had a response. While these two studies established that poorly differentiated NENs can be sensitive to platinum-based chemotherapy, prognosis remained poor with less than 20% of patients surviving longer than 2 years. This experience has been validated in multiple clinical studies: 14 of them are reviewed in Table 1 including 5 prospective and 9 retrospective trials. In summary, RR ranged from 14 to 67%, and overall survival (OS) remained poor between 6 and 22 months [10,11,24,37–49].

**Table 1:** Efficacy of platinum-based chemotherapy for neuroendocrine carcinoma

Reference	Study design	NEC study population, *n* patients	Intervention	ORR	Median PFS, months	Median OS, months
Moertel et al. 1991 [10]	Prospective, observational	18 anaplastic NEC	Cisplatin/etoposide	67%	8	19
Hainsworth et al. 2006 [43]	Prospective, phase II, single arm	15 extrapulmonary poorly differentiated NEC	Carboplatin/etoposide + paclitaxel	53%	8	15
Li et al. 2017 [44]	Prospective, phase II, Single arm, open label	40 GEP-NEC	Cisplatin/irinotecan + octreotide LAR	NA	6	13
Walter et al. 2017 [45]	Prospective cohort	202 GEP-NEC	Carboplatin/etoposide or cisplatin/etoposide	50%	6	12
Zhang et al. 2020 [46]	Prospective, phase II, randomised	66 GEP-NEC	Cisplatin/etoposide versus cisplatin/irinotecan	42%	6 (EP) 6 (IP)	11 (EP) 10 (IP)
Mitry et al. 1999 [11]	Retrospective	41 PD-NEC	Cisplatin/etoposide	42%	9	15
Iwasa et al. 2010 [41]	Retrospective	21 GEP-NEC	Cisplatin/etoposide	14%	2	6
Okita et al. 2011 [40]	Retrospective	22 gastric-NEC	Cisplatin/irinotecan	75%	7	22
Nakano et al. 2012 [48]	Retrospective	44 extrapulmonary poorly differentiated NEC	Cisplatin/irinotecan	64%	7	16
Yamaguchi et al. 2014 [37]	Retrospective	206 GEP-NEC	160 cisplatin/irinotecan 46 cisplatin/etoposide	50% (IP) 28% (EP)	5 (IP) 4 (EP)	13 (IP) 7 (EP)
Heetfeld et al. 2015 [24]	Retrospective	113 GEP-NEC	Platinum/etoposide	35%	5	16
Imai et al. 2016 [38]	Retrospective	19 GEP-NEC	Carboplatin/etoposide	47%	7	13
Raj et al. 2017 [39]	Retrospective	25 PD-NEC	Platinum agents	37%	NA	10
Brandi et al. 2018 [42]	Retrospective	21 GEP-NEC and CUP-NEC	Platinum/etoposide	52%	7	16

*CUP* cancer of unknown primary, *EP* platinum/etoposide, *GEP* gastroenteropancreatic, *GI* gastrointestinal, *IP* platinum/irinotecan, *NA* not available, *ORR* objective response rate, *OS* overall survival, *PD* poorly differentiated, *PFS* progression-free survival

In addition to well-differentiated morphology, a lower Ki-67 index has also been associated with a lower efficacy in terms of RR. In the NORDIC NEC study [9], patients with NEC G3 (WHO 2010) who received first-line platinum-based chemotherapy were reported to have a 31% RR to first-line chemotherapy. Patients with Ki-67 below 55% were found to have a worse RR compared to those with a Ki-67 ≥55% (15% versus 42%). The relationship between tumour response to Ki-67 index and morphology was further validated in a retrospective study from eight European centres [24] that assessed the effect of carboplatin/etoposide in patients with NET G3 (*n*=37) and NEC (*n*=167). Disease control rate (DCR) and progression-free survival (PFS) were found to be significantly higher in NEC in comparison to NET G3, whereas OS was shown to be significantly longer in NET G3 (99 versus 17 months). Additional biomarkers for response to platinum-based chemotherapy have been evaluated. A multicentre retrospective study [31] investigated a population of 70 patients with pancreatic NENs G3 that had received platinum-based chemotherapy and correlated outcomes to clinicopathological and molecular features. Patients with *RB* loss or *KRAS* mutation showed a high RR, 80% and 77%, respectively. In the patients with pancreatic NET G3, there were no tumours with *RB* loss or *KRAS* mutation, and the RR were 24% and 23%, respectively. This suggests it may be valuable to use these immunohistochemical and genetic markers in future pancreatic NEC classifications.

Randomised studies comparing alternatives to cisplatin/etoposide are lacking with one exception. In a randomised phase II study [46], the efficacy of cisplatin/etoposide was compared to cisplatin/irinotecan for the first-line therapy in patients with advanced GEP-NEC. Sixty-six patients were randomised to either cisplatin/etoposide or cisplatin/irinotecan. Primary endpoint was objective response rate (ORR), and the study was powered to detect a 30% RR in the cisplatin/etoposide arm and 50% RR in the experimental cisplatin/irinotecan arm. This primary endpoint was not met as ORR was 42.4% in both study arms. In the cisplatin/etoposide arm, median PFS was 6.4 months and median OS was 11.3, with similar outcomes in the cisplatin/irinotecan arm, 5.8 months for PFS and 10.2 months for OS, respectively. One encouraging note is that there are several ongoing clinical trials studying different strategies for first-line therapy for GEP-NEC patients (Table 2). We wish to highlight a phase II study investigating the efficacy of a platinum agent in combination with anti-programmed death 1 (PD-1) antibody (NCT03980925) and a phase II study analysing 177Lu-DOTATATE plus anti PD-1 antibody (NCT04525638), in patients where at least one tumour lesion should have expression of somatostatin receptors confirmed by PET. Other ongoing studies include a phase II study examining platinum-agents plus everolimus (NCT02695459), and two ongoing randomised phase II studies comparing the efficacy of platinum agents to capecitabine/temozolomide (NCT02595424), as well as modified FOLFIRINOX (folinic acid, fluorouracil, irinotecan and oxaliplatin) (NCT04325425).

**Table 2:** Randomised clinical trials currently recruiting patients with neuroendocrine carcinoma

Study abbreviation	ClinicalTrials.gov reference	Trial design	Intervention(s)	Primary endpoint
NIPINEC	NCT03591731	Phase II, randomised	Arm A: nivolumab Arm B: nivolumab/ipilimumab	ORR
FOLFIRINEC	NCT04325425	Phase II, randomised	Experimental: modified FOLFIRINOX Comparator: platinum/etoposide	Median PFS
SENECA	NCT03387592	Phase II, randomised	Experimental: capecitabine and temozolomide Comparator: FOLFIRI	1: DCR 2: treatment related adverse events
BEVANEC	NCT02820857	Phase II, randomised	Experimental: FOLFIRI/ bevacizumab Comparator: FOLFIRI	Proportion of patients alive
NET02	NCT03837977	Phase II, randomised	Arm A: liposomal irinotecan, fluorouracil, folinic acid Arm B**:** docetaxel	PFS
NA	NCT02687958	Phase II, randomised	Experimental: everolimus Non-intervention: observational	PFS
NA	NCT02595424	Phase II, randomised	Experimental: capecitabine + temozolomide Comparator: cisplatin/etoposide or carboplatin/etoposide	PFS

*DCR* disease control rate, *FOLFIRI* leucovorin, fluorouracil, and irinotecan, *ORR* objective response rate, *PFS* progression-free survival, *NA* not available

### Second-line setting

There is currently no consensus regarding optimal therapeutic strategy after first-line therapy in advanced GEP-NEC patients [13]. As a consequence, patients have received heterogeneous treatment regimens (Table 3). Second-line treatments that are recommended by ESMO and ENETS [12••,^13^] include fluorouracil-based chemotherapy in combination with either irinotecan or oxaliplatin as well as temozolomide in monotherapy or in combination with capecitabine.

**Table 3:** Studies on second- and third-line therapies for neuroendocrine carcinoma

Reference	Study design	NEC study population, *n* patients	Intervention	ORR	Median PFS, months	Median OS, months
Chen et al. 2020 [55]	Open-Label, multicentre, phase II, single arm study	22 GEP-NEC, 2^nd^-line	TLC388	0%	2	4
Welin et al. 2011 [50]	Retrospective, observational	25 poorly differentiated endocrine carcinoma, 2^nd^-line	Temozolomide, +/- capecitabine, +/- bevacizumab	33%	6	22
Hentic et al. 2012 [53]	Retrospective	19 NEC, 2^nd^-line	FOLFIRI	31%	4	18
Olsen et al. 2012 [51]	Retrospective	28 GEP-NEC, 2^nd^/3^rd^-line	Temozolomide	38%	2	4
Du et al. 2013 [49]	Retrospective	11 GI-NEC, 1^st^-line	FOLFIRI	64%	7	13
Ando et al. 2015 [59]	Retrospective, observational	10 GEP-NEC, 2^nd^-line	Amrubicin	20%	3	5
Nio et al. 2015 [57]	Retrospective, observational	13 GEP-NEC, 2^nd^-line	Amrubicin	40%	4	7
Hadoux et al. 2015 [54]	Retrospective, observational	12 GEP-NEC, 8 other NEC, 2^nd^/3^rd^-line	FOLFOX	NA	5	10
Apostilidis et al. 2016 [56]	Retrospective, observational	30 extrapulmonary poorly differentiated NEC, 2^nd^-line	Topotecan	NA	2	4
Araki et al. 2016 [58]	Retrospective	18 extrapulmonary PD-NEC, GI-NEC, 1^st^/2^nd^-line	Amrubicin	19%	4	8
Rogowski et al. 2019 [52]	Retrospective	12 NEC, 2^nd^-line	Temozolomide/capecitabine	NA	3	5
Sugiyama et al. 2020 [90]	Retrospective, observational	5 GI-NEC, 2^nd^-line	FOLFIRI	40%	6	11

TLC388 is a camptothecin derivate targeting topoisomerase I. *FOLFIRI* folinic acid, 5-fluorouracil, irinotecan, *FOLFOX* folinic acid, 5-fluorouracil, oxaliplatin, *GEP* gastroenteropancreatic, *GI* gastrointestinal, *NA* not available, *ORR* objective response rate, *OS* overall survival, *PFS* progression-free survival

In a retrospective study from 2011 [50], efficacy of temozolomide-based chemotherapy in second-line/third-line was studied among 25 patients with poorly differentiated endocrine carcinoma (WHO 2000). Ki-67 index ranged from >20 to 90%, and tumours were mostly of gastrointestinal origin. Median PFS was 6 months and median OS was 22 months. The study also found that non-responders to first-line chemotherapy, with a Ki-67 below 60% and uptake on somatostatin receptor scintigraphy, had a better response to temozolomide-based chemotherapy. Temozolomide monotherapy was further examined in the second- or third-line setting in 28 NEC patients [51]. Seven had pancreatic NEC and showed a median OS of 7.0 months versus 21 patients with non-pancreatic NEC that had a median OS of 2.9 months. Median PFS was 3.3 months versus 1.9 months, respectively. Only 16 were evaluable for radiological response and DCR was 38%, while no PR or CR were observed. Efficacy of temozolomide plus capecitabine was further assessed [52] in patients with GEP-NET G3 (*n*=20) and GEP-NEC G3 (*n*=12). DCR was 70% in the NET group and 30% in NEC group. Median PFS was 15.3 months for NET G3 and 3.3 months for NEC G3, whereas median OS was 22 months and 4.6 months, respectively.

Fluorouracil-based chemotherapy regimens have also been investigated. Hentic et al. [53] reported in a retrospective study the efficacy of the FOLFIRI regimen (folinic acid, fluorouracil, and irinotecan) among 39 patients with gastrointestinal NEC. Only 19 patients completed the treatment course, the rest could not follow through due to toxicity or death. The median OS among these 19 patients was 18 months and median PFS 4.0 months.

Du et al. [49] studied the efficacy of FOLFIRI in 11 patients with gastrointestinal-NEC, the median PFS was 6.5, and the median OS was 13.0 months. Furthermore, a single-centre retrospective study from 2015 analysed the antitumour efficacy of FOLFOX (folinic acid, fluorouracil, and oxaliplatin) among 20 patients with NEC after progression on platinum-based regimens. The median follow-up was 19 months and median PFS was 4.5 months. Among the 17 evaluable patients, 5 PR (29%), 6 stable diseases (SD) (35%), and 6 progressive diseases (PD) (35%) were observed. Median OS was 9.9 months [54]. In the study by Heetfeld et al. [24], the effects of second- (*n*=79) and third-line (*n*=39) FOLFIRI or FOLFOX among patients with NEC were also studied. Median PFS for second-line was 3.0 months and median OS 7.0 months, for third-line median PFS, and median OS was 2.5 and 6.2 months.

Topoisomerase inhibitors were traditionally considered an alternative treatment strategy in small-cell lung cancer and was therefore subject to one prospective [55] and four retrospective [56–59] studies in NEC patients. Median PFS was about 2–4 months with a median OS of between 4 and 7 months and a RR between 19 and 40%. This class of drugs is currently not recommended in neither ENETS nor ESMO guidelines.

## Future treatment strategies

Numerous agents have been investigated for treatment of NEC throughout the years, supported either by a biological rationale or efficacy in other relevant diseases. Encouragingly, the current clinical trial pipeline also shows a high activity. Here we have focused on three different classes of drugs believed to provide promising new alternatives: 177Lu-DOTATATE, immunotherapy, and anti-*BRAF* treatment.

A study published in 2018 analysed the treatment outcomes after 177Lu-DOTATATE in patients with a GEP-NEN having a high Ki-67 index [60]. Thirty-three patients with advanced GEP-NENs (Ki-67 15–70%) displaying adequate uptake on somatostatin receptor imaging received treatment with 177Lu-DOTATATE, 6% achieved PR, while 64% showed SD. The median PFS was 23 months, and the median OS was 52.9 months. Twenty-three patients had Ki-67 <35% and a median PFS of 26.3 months, while 10 patients had Ki-67 >35% and a median PFS of only 6.8 months. A few more studies show almost similar outcomes with RR of about 42% in subgroup analyses for GEP-NENs with Ki-67 ≥55% [61–63]. As previously mentioned, Lu177-DOTATATE plus nivolumab is now subject to a phase II study in the first-line setting (NCT04525638).

Translational studies demonstrated a rationale for immunotherapy in GEP-NECs by displaying a different microenvironment compared with well-differentiated GEP-NETs [64]. NECs commonly showed presence of cell surface receptor PD-1, programmed death-ligand 1, and/or tumour-infiltrating lymphocytes (TILs), which were also poor prognostic factors [65–68]. At the same time, immunotherapeutic agents were approved for diseases with similar biology to NEC including avelumab for treatment of Merkel cell carcinoma [69–71], as well as durvalumab and atezolizumab for treatment of small-cell lung cancer [72–75]. Based on the results from a phase I/II, non-randomised and randomised multicentre trial, nivolumab has been FDA approved as third-line monotherapy for metastatic SCLC [76].

In a controlled, phase III trial, patients with extensive stage small-cell lung cancer were randomised to carboplatin/etoposide with either atezolizumab or placebo. Hazard ratio for death was favouring the combination therapy, 0.70 (95% confidence interval 0.54–0.91) [73]. This established a rationale for combining chemotherapy and immunotherapy using checkpoint inhibitors also in NEC.

Recent studies reporting on the efficacy of immunotherapy in NEC patients include two phase II studies performed by Vijayvergia et al. on pembrolizumab in 29 patients with NEN G3 [77•]. Of these, 19 (66%) had poorly differentiated NEC and nine (34%) had well-differentiated NET. Twenty-eight (97%) had prior platinum-based chemotherapy. There was one (3.4%) PR and six (20.7%) SD, and 17 (58.6%) had PD. Median OS was 20.4 weeks. While ten tumour samples (67%) stained positive for PD-1 and eight tumour samples (53%) had infiltrating TILs, these biomarkers were not correlated with DCR. In a second phase II study [78], avelumab was studied in 29 patients with advanced GEP-NECs. Interim analysis after 8 weeks showed a DCR of 32% (4 SD, 2 PR). In responders, mean duration of disease control was 20 (±13.8) weeks, with 4 patients showing SD or PR ≥6 months. Median OS was 4.2 months.

Patel et al. [79] reported data from a phase II basket trial of anti-CTLA-4 and anti-PD-1 antibodies with results on non-pancreatic NENs presented separately. Thirty-two patients were included and 18 (56%) were high-grade NEN. One patient (3%) had CR and seven (22%) had PR. All responses were observed in the NEC cohort, with ORR of 44% for high-grade NEC (WHO 2010). Similarly, Klein et al. [80] reported data from a subgroup of advanced NENs included in the CA209-538 clinical trial on ipilimumab and nivolumab. There were 29 patients included, of which 13 (45%) had high-grade tumours. Seven patients had PR and the ORR was 24%. Objective responses were achieved in seven intermediate and high-grade tumours, two were NEC.

While combination immunotherapy is considered a promising new treatment strategy for NEC, monotherapy with either anti PD-1 or anti PD-L1 antibodies appears to be less favourable. In a phase II, multicentre, single-arm study, the efficacy of spartalizumab was studied in metastatic well-differentiated NETs (*n*=95) and poorly differentiated GEP-NECs (*n*=21). The ORRs were 7.4% in the NET group and 4.8% in the GEP-NEC group. Patients with PD-L1 expressions of more than 1% or presenting with more than 1% of CD8+ cells at baseline were observed to have higher ORR. The primary endpoint was not met [81]. Clarifying results from clinical trials are awaited (Table 2) concerning the efficacy of immunotherapy in NEC patients.

Finally, anti-*BRAF* therapy has shown to be efficient across different cancers harbouring *BRAF* V600E mutations [82–85]. However, some diseases, most notably colorectal cancer, have not shown a high level of responsiveness to such drugs [85,86]. The results from three separate studies revealed that 22% (39/174) of colorectal NECs harboured a *BRAF* V600E alteration [87, 88, 89•]. In their translational study, Capdevila et al. reported that *BRAF* V600E mutant colon NEC may benefit from *BRAF* inhibition in monotherapy and that *EGFR* status is essential to predict innate sensitivity and acquired resistance by a differential methylation of its gene regulatory regions [89•]. While these are promising preclinical data, the field still awaits clarifying results, whether anti*-BRAF* therapy could provide relief to patients with *BRAF* V600E mutated colon NECs. Klempner [87] described two patients with colorectal NEC treated with dabrafenib (*BRAF* inhibitor) and trametinib (*MEK* inhibitor). Both achieved PR that were ongoing at 7 and 9 months, respectively. Burkart [14] presented two cases with colorectal NEC, patient one was treated with dabrafenib and trametinib and achieved PR, lasting for 5 months, whereas the second patient who received dabrafenib and pazopanib (broad tyrosine kinase inhibitor) had achieved disease stabilisation, with progression after 6 months. Finally, in a basket trial including various cancers harbouring oncogenic *BRAF* mutations receiving vemurafenib, two patients with NEC were described. One had a PR lasting 4 months and the second had SD of unknown duration [15].

## Summary

A majority of the studies underlying current treatment recommendations for GEP-NECs have a retrospective and observational design and examined small patient populations. Based on the available data, ESMO and ENETS guidelines [12••,^13^] recommend platinum-based chemotherapy for patients with advanced GEP-NEC. For second-line treatment, the guidelines recommend fluorouracil- or temozolomide-based chemotherapy. The increased awareness of the molecular heterogeneity of these tumours might suggest that it will be more beneficial for future treatment regimens to be individualised and profiled towards biomarkers. There is an urgent need for larger prospective clinical trials of comparative nature, looking at new promising chemotherapeutic and immunotherapeutic regimens as well as targeted gene therapy.
